# The role of KIF14 in patient-derived primary cultures of high-grade serous ovarian cancer cells

**DOI:** 10.1186/s13048-014-0123-1

**Published:** 2014-12-21

**Authors:** Brigitte L Thériault, Paulina Cybulska, Patricia A Shaw, Brenda L Gallie, Marcus Q Bernardini

**Affiliations:** Campbell Family Cancer Research Institute, Ontario Cancer Institute, University Health Network, Toronto, ON Canada; Department of Obstetrics and Gynecology, University of Toronto, Toronto, ON Canada; Division of Gynecological Oncology, University Health Network, Toronto, ON Canada; Department of Pathology, University Health Network, Toronto, ON Canada; Princess Margaret Hospital, University Health Network Tissue Bank, Toronto, ON Canada; Division of Visual Science, Toronto Western Hospital Research Institute, Toronto, ON Canada; Departments of Medical Biophysics, Molecular Genetics, and Ophthalmology, University of Toronto, Toronto, ON Canada; Princess Margaret Cancer Centre, Rm M700, 610 University Ave, Toronto, Ontario M5G 2M9 Canada

**Keywords:** KIF14, Ovarian tumor tissue, Primary culture, shRNA, Proliferation, Colony formation

## Abstract

**Objective:**

Previously, it has been shown that *KIF14* mRNA is overexpressed in ovarian cancer (OvCa), regardless of histological subtype. *KIF14* levels are independently predictive of poor outcome and increased rates of recurrence in serous OvCa patients. Furthermore, it has been shown that KIF14 also controls the *in vivo* tumorigenicity of OvCa cell lines. In this study, we evaluate the potential of KIF14 as a therapeutic target through selective inhibition of KIF14 in primary high-grade serous patient-derived OvCa cells.

**Methods:**

To assess the dependence of primary serous OvCa cultures on *KIF14*, protein levels in 11 prospective high grade serous ovarian cancer samples were increased (*KIF14* overexpression by transfection) or decreased (anti-*KIF14* shRNA) *in vitro*, and proliferative capacity, anchorage independence and xenograft growth were assessed.

**Results:**

Seven of eleven samples demonstrated increased/decreased *in vitro* proliferation in response to KIF14 overexpression/knockdown, respectively. When examining *in vitro* tumorigenicity (colony formation) and *in vivo* growth (subcutaneous xenografts) in response to *KIF14* manipulation, none of the samples demonstrated growth in soft agar (11 samples), or xenograft growth (4 samples).

**Conclusions:**

Although primary high-grade serous OvCa cells may depend on KIF14 for *in vitro* proliferation we were unable to demonstrate a role for KIF14 on tumorigenicity or develop an *in vivo* model for assessment. We have, however developed an effective *in vitro* method to evaluate the effect of target gene manipulation on the proliferative capacity of primary OvCa cultures.

**Electronic supplementary material:**

The online version of this article (doi:10.1186/s13048-014-0123-1) contains supplementary material, which is available to authorized users.

## Introduction

Located on chromosome 1q32, KIF14 has been demonstrated to be overexpressed at the genomic and gene expression levels in multiple cancers, including breast and retinoblastoma [1], liver [2], renal papillary [3], lung [4], and ovarian cancers (OvCas) [1,5-11]. Comparative genomic hybridization (CGH) studies determined in breast, ovarian, prostate, oesophageal, multiple myeloma and hepatocellular carcinomas that gain in chromosome 1q32 correlated highly with recurrence and poor differentiation, and this gain has been postulated as an early or initiating event [12-18].

KIF14 has been shown to be essential for the final phase of cytokinesis [19,20]. A molecular motor and microtubule-associated protein, KIF14 was shown to interact directly with Protein regulating cytokinesis 1 (PRC1) and Citron kinase (CIT), displaying a central organizing role in cytokinesis [19]. High *KIF14* mRNA expression is documented in many cancers including hepatocellular [2,21,22] and laryngeal carcinomas [23], while *KIF14* expression levels correlate with adverse features in papillary renal tumors [3] and pancreatic carcinomas [24]. Furthermore, KIF14 expression has been associated with chemoresistance in triple-negative breast cancers [25,26]. We have previously shown prognostic significance of *KIF14* mRNA in breast, lung and ovarian cancers [5,27,28] and uncovered transcriptional and epigenetic regulation of *KIF14* overexpression in ovarian cancers [29].

*KIF14* is overexpressed in the majority of primary OvCa tumors regardless of stage. Close to 30% of serous OvCas displayed genomic gain of *KIF14* that correlates with high *KIF14* overexpression, suggesting that *KIF14* gain, when present, may be an early event in the development of serous OvCa [5]. *KIF14*^LOW^ serous patients demonstrate a significant survival advantage over *KIF14*^HIGH^ patients enforcing the predictive value of *KIF14* expression for outcome of serous OvCa patients [5]. Previously, it has been shown that overexpression of *KIF14* in OvCa cell lines significantly increased proliferation and the number of soft agar colonies. *KIF14* knockdown in the same immortalized cell lines showed reduced proliferation, increased apoptosis, and most importantly, significantly reduced colony formation to a greater extent than proliferation [5].

Since *KIF14* displays a favourable therapeutic ratio (very low expression in adult tissues), knowledge of the exact function(s) of KIF14 in the progression of OvCa may define an important therapeutic target. To conclusively demonstrate the importance of KIF14 in promoting OvCa and to develop *KIF14* overexpression as a “high risk” marker for OvCa, the next step was to study the implications of *KIF14* expression in the progression of primary OvCa tumors derived from patients. In this study, we evaluated the potential of selective *KIF14* overexpression or inhibition in primary high-grade serous patient-derived OvCa cells.

## Materials and methods

### Clinical samples

Thirty fresh high-grade serous ovarian tumor samples were obtained from OvCa patients admitted to the Gynecological Oncology Clinic, Princess Margaret Hospital, Toronto, ON. Tumor samples (or ascites fluid) were collected at initial debulking surgery, prior to administration of chemotherapy, and released by the University Health Network (UHN) Biobank, Toronto, ON. The UHN Research Ethics Board approved this study, and all tissues were banked with written informed consent. All UHN Biobank tissues (adjacent H and E stained slides) were reviewed by a gynecologic pathologist (PAS) to ensure that released tissues were of high-grade serous histology, and contained >80% tumor cells.

### Primary derivative cultures of OvCa tissues

Samples destined for derivative cultures were immediately taken to the laboratory where a small portion was reserved for RNA and DNA isolation, and the remainder cultured as previously described and commonly performed in our laboratory [30]. Early-passage cells (passages 2–5) were employed for our analyses, to avoid the induction of genomic changes due to culture adaptation. This culture time was sufficient to obtain cells for *KIF14* manipulations (yield of several million cells within 2–3 passages) [30,31]. We obtained 29 primary samples from the UHN Biobank. From these, we were able to develop short-term *in vitro* derivative cultures from 11 tumor samples.

### RNA extraction and reverse transcription

Total RNA was extracted from primary tissues and cells by homogenizing tissue through a syringe in TRIzol reagent (Invitrogen, Mississauga, ON), followed by chloroform extraction and isopropanol and ethanol precipitations. One microgram of total RNA was used in reverse transcription (RT) reactions, as previously described [5]. To confirm RT, 1 L of each reaction was tested in endpoint PCR for KIF14 and the housekeeping gene HPRT (hypoxanthine phosphoribosyl transferase) as described [5].

### End-point and real-time PCR

For end-point PCR, 1 μL of the RT reaction was added to a 25 μL PCR reaction containing 0.5 U Hot Start Taq Polymerase (Fermentas, Burlington, ON), 0.2 mM dNTPs, 1.5 mM MgCl_2_ and KIF14 primers; cycling conditions were previously described [5]. TBP was used as an endogenous control, and products were visualized by gel electrophoresis and ethidium bromide staining.

For real-time PCR, RT reaction products were diluted 10-fold with RNAse/DNAse-free ddH_2_O, and 1.5 L was added to 1X TaqMan PCR master mix (Applied Biosystems, Life Technologies, Carlsbad, CA) and 1X TaqMan Gene Expression Assay primer-probe mix for KIF14 (Hs00978216_m1). Mean expression of three housekeeping genes was used as an endogenous control: TBT (Tata-box binding protein, Hs_99999910_m1), HPRT (Hypoxanthine phosphoribosyl transferase, Hs_99999909_m1), and GAPDH (glyceraldehyde-3-phosphate dehydrogenase, Hs_99999905_m1), due to their stable expression in primary OvCa tumor tissues [5]. Triplicate reactions were conducted for each gene and each tissue sample, and PCR performed using the SDS 7900HT system as described [5]. SDS 2.1 Software (ABI) was used to calculate Ct relative expression values, normalized to endogenous control genes, and relative to either untreated, scrambled or empty-vector controls.

### Established cell line culture, shRNA lentivirus construction, transductions and transfections

SKOV3 and OvCa429 cells (a kind gift from Dr. Mark Nachtigal, University of Manitoba, Winnipeg, MB) were grown in DMEM H16 minimal medium (SKOV3) or alpha-MEM (OvCa 429) supplemented with penicillin-streptomycin and 10% fetal bovine serum at 37°C, 5% CO_2_ in a humidified chamber. Packaging cells (293FT, Invitrogen) were grown as SKOV3 cells. All parental and derived stable cell lines were authenticated using STR (short tandem repeat) profiling (The Centre for Applied Genomics, Hospital for Sick Children, Toronto, ON). Anti-KIF14 shRNA lentiviruses were generated by co-transfection of pLKO.1 containing shRNAs and expressing puromycin resistance, targeting human *KIF14* (5 separate constructs, #816-819 plus a Scrambled control; Sigma-Aldrich) with packaging constructs pPAX2 and pMD2G (a kind gift from Dr. Jason Moffat, University of Toronto, Toronto, ON) in 293FT cells using Gene Juice (EMD Biosciences, Gibbstown, NJ) according to manufacturer instructions. Virus supernatant was harvested 48 h post-transfection and concentrated 10-fold using the LentiX viral concentrator solution (Clontech Laboratories, Mountain View, CA). *KIF14* knockdown in primary OvCa cultures and cell lines was confirmed 72 h post-transduction using real-time PCR and western blot (WB) analyses. Overexpression of *KIF14* was generated by transfection of KIF14-EGFP (a kind gift from Dr. Francis Barr, Max Planck Institute of Biochemistry, Martinsreid, Germany) or pcDNA 3.1 expressing neomycin resistance (empty vector control). Transfections were conducted with Turbofect in vivo transfection reagent (Fermentas) according to manufacturer’s instructions. WB analysis was performed by probing with a polyclonal rabbit anti-KIF14 antibody (1:500, Bethyl Laboratories, Montgomery, TX) or anti-EGFP antibodies (1:2000, Abcam, Cambridge, MA) normalized to β-tubulin (1:1000, Sigma-Aldrich). Horseradish peroxidase-labeled secondary antibodies (1:10,000, Chemicon, Billerica, MA) were detected using a chemiluminescence reagent (Denville Scientific, Metuchen, NJ) and incubated with photographic film (Denville).

### Immunofluorescence

Cells were stained with a polyclonal rabbit anti-KIF14 antibody (Bethyl Laboratories), followed by a mouse anti-rabbit Alexa 488 secondary antibody (Molecular Probes, Mississauga, ON), and nuclei visualized by DAPI staining (Sigma-Aldrich). Stained cells were visualized at a magnification of up to 400X with an epifluorescence microscope (Leica, Wetzlar, Germany).

### Proliferation and soft-agar colony forming assays

Proliferation was measured by surrogate intracellular ATP readings using a commercially available assay kit (Cell TiterGlo; Promega) according to manufacturer’s instructions. Cells were seeded in triplicate at 5000 cells/96-well dish (Day 0) and counted every 2 to 3 days for up to 12 days. Colony assays were conducted by seeding cells in triplicate at 1 × 10^4^ cells (primary OvCa cells) or 1 × 10^3^ cells (OvCa cell lines)/6-well dish in 0.3% noble agar, atop a plug of 0.6% noble agar in growth medium (Biorad Laboratories, Mississauga, ON). After 14 (OvCa cell lines) or 30 days of growth, colonies were stained with crystal violet and counted with the 1.5X objective of a dissecting microscope (Leica). All experiments were conducted on three separate occasions in triplicate.

### Subcutaneous xenografts

Female NOD-SCID gamma mice were injected subcutaneously into the flank with 1 million cells mixed at a 1:1 ratio with Matrigel (BD Biosciences, Mississauga, ON) in a 200 L total volume. Four primary OvCa samples that were manipulated for either overexpression or knockdown, plus their respective control cells were injected into triplicate mice, and monitored for a total of 6 months for appearance of tumors. These mice were studied using protocols approved by the Animal Care Committee of the Ontario Cancer Institute, in accordance with Canadian Council on Animal Care guidelines.

### Statistical analyses

Unpaired t-tests were performed to determine statistical differences (where significance < 0.05) between cell number in response to *KIF14* manipulation (overexpression or knockdown) relative to the control cells (empty vector or scrambled control) using Graph Pad Prism 4.0.

## Results

### Short-term culture of primary high-grade serous OvCa cells reduces endogenous KIF14 levels

We obtained 29 high-grade serous primary samples from the UHN Biobank. From these, we were able to develop short-term *in vitro* derivative cultures from 11 tumor samples. These 11 samples were then further characterized for *KIF14* expression.

**Figure 1:**
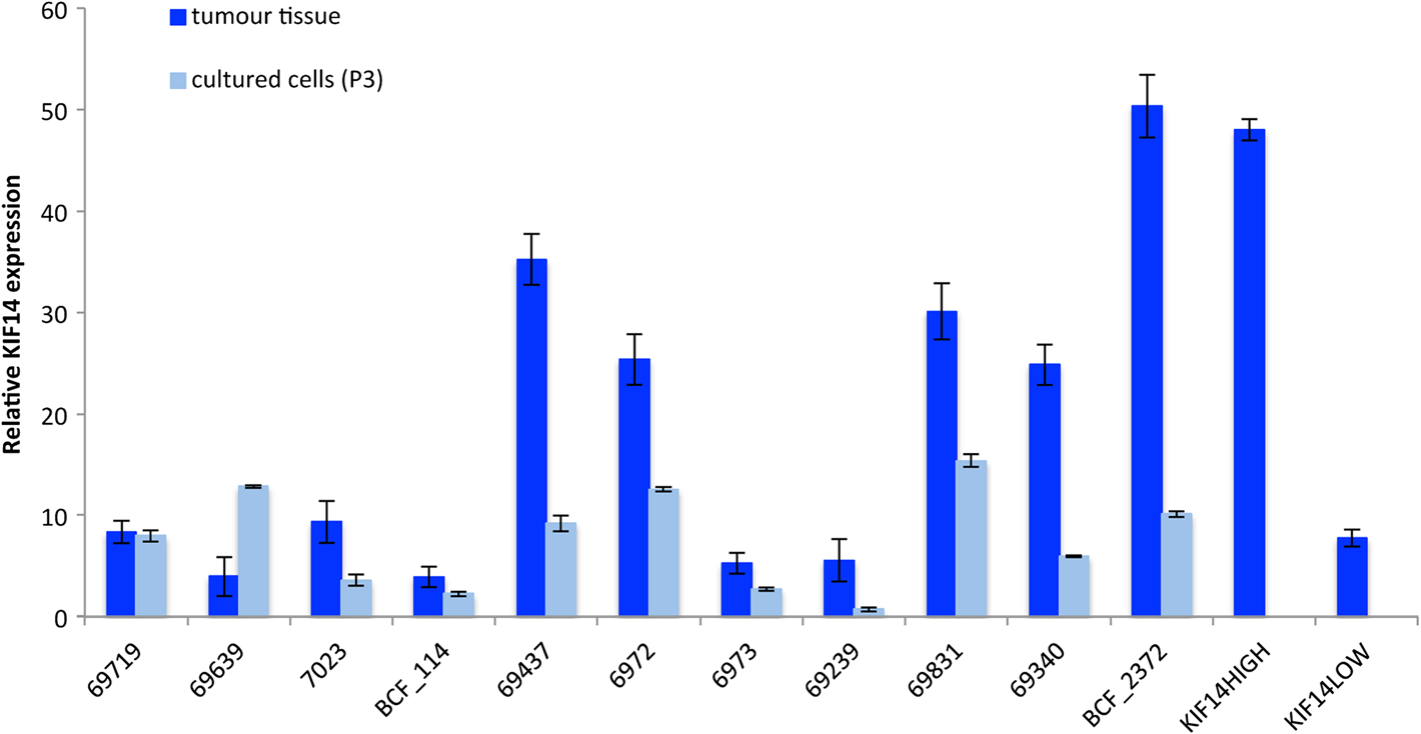
***KIF14*** **mRNA expression in response to short-term** ***in vitro*** **culture conditions.** Eleven primary OvCa samples (high-grade serous histology) were measured for *KIF14* expression in the primary tissue (dark blue) and in short-term culture (passage 3, light blue). Previously characterized *KIF14* ^*HIGH*^ and *KIF14* ^*LOW*^ primary OvCa samples (5) were included as controls. Error bars represent standard deviation of 3 independent measurements for each sample.

To determine whether the expression of *KIF14* varied with adaptation to culture, *KIF14* mRNA was measured in primary tissues and short-term primary OvCa cultures (passage 3) using real-time PCR. As depicted in Figure 1, 45% of the primary tumor tissues tested (5 out of 11; dark blue) exhibit very high *KIF14* expression in comparison with previously characterized *KIF14*^*HIGH*^ and *KIF14*^*LOW*^ primary OvCa tumors [5], and would belong to the *KIF14*^*HIGH*^ expressers group, while the remaining 55% of samples (6 out of 11) would be grouped into the *KIF14*^*LOW*^ expressers group. For most primary samples however (9 out of 11), short-term adaptation to culture (approximately 3 weeks) did significantly decrease *KIF14* expression (2 to 5-fold decrease; Figure 1). One sample (69719) did not exhibit any change in *KIF14* expression, while one sample (69639) showed an increase in *KIF14* expression in response to *in vitro* culture conditions.

### Primary high-grade serous OvCa cells depend upon KIF14 expression for proliferation

**Figure 2:**
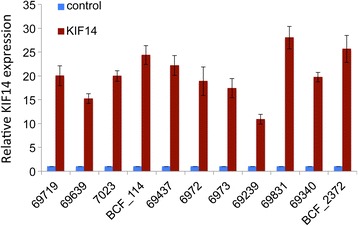
***KIF14*** **knockdown in primary OvCa samples.** Eleven primary OvCa samples were transduced with lentivirus expressing anti-*KIF14* shRNA (LV-816) (green), and mRNA expression measured 21 days (passage 5) post-transduction in comparison to a scrambled shRNA control (control; blue). *KIF14* expression in LV-816 cells normalized to control cell expression (set as 1). Error bars represent standard deviation of three independent experiments.

To test whether primary OvCa cells are dependent upon *KIF14* expression for their tumorigenicity, 11 samples were transduced with an anti-*KIF14* shRNA lentivirus (LV-816 plus a scrambled control). We observed expression of KIF14 protein via immunofluorescence (Additional file 1: Figure S1); a significant decrease in KIF14 protein expression is seen after 14 days post-transduction (3 passages) in most cells. *KIF14* mRNA expression was also measured in these samples after 21 days (5 passages) of culture via real-time PCR. A significant decrease in *KIF14* expression was seen in all transduced samples (2 to 5-fold, Figure 2).

**Figure 3:**
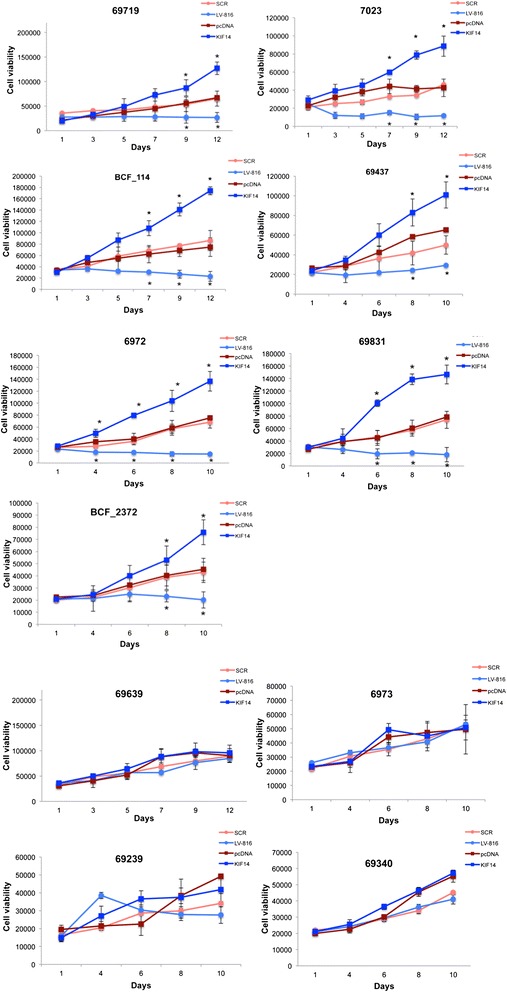
**The effect of KIF14 overexpression/knockdown on primary OvCa cultures.** Cell viability readings in response to *KIF14* overexpression (KIF14, dark blue) or *KIF14* knockdown (LV-816, light blue) relative to their respective controls (pcDNA (KIF14), dark red; SCR (LV-816), light red). *P <0.05. Error bars represent standard deviation of 3 independent experiments.

The growth properties of these 11 primary OvCa samples were measured in response to *KIF14* knockdown via cellular ATP. Most samples (7 out of 11, 64%) demonstrated a significant decrease in cell proliferation in response to *KIF14* knockdown (P < 0.05; Figure 3). However, a smaller percentage of samples (4 out of 11, 36%) showed no significant change in proliferation even when KIF14 was reduced (P > 0.01; Figure 3).

### Overexpression of KIF14 increases the proliferative capacity of primary high-grade serous OvCa cells

**Figure 4:**
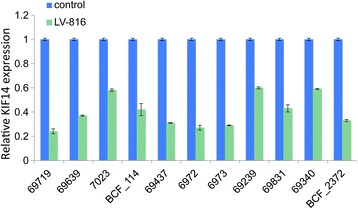
***KIF14*** **overexpression in primary OvCa samples.** Eleven primary OvCa samples were transiently transfected with KIF14-EGFP (red), and mRNA expression measured 21 days (passage 5) post-transfection in comparison to the empty vector control (pcDNA; blue). *KIF14* expression in KIF14-EGFP cells normalized to control cell expression (set as 1). Error bars represent standard deviation of three independent experiments.

To study the consequence of *KIF14* overexpression on the tumorigenic phenotype of closely derived patient samples, short-term cultures *KIF14*^HIGH^ or *KIF14*^LOW^ OvCa cells were engineered to overexpress *KIF14* via transient transfection of an EGFP-tagged *KIF14* cDNA plasmid [19]. We observed strong expression of KIF14-EGFP in most cells after 14 days post-transfection (3 passages; Additional file 2: Figure S2). Overexpression of *KIF14* mRNA was also seen in all samples for at least 21 days post-transfection (Figure 4).

We measured the growth properties of these 11 primary OvCa samples in response to *KIF14* overexpression via cell proliferation assays. The majority of samples (7 out of 11, 64%) demonstrated a significant increase in cell proliferation in response to *KIF14* overexpression (P < 0.05; Figure 3), while a smaller percentage of samples (4 out of 11, 36%) showed no significant change in proliferation (P > 0.01; Figure 3).

### Survival of primary high-grade serous OvCa cells is impaired under in vitro or in vivo anchorage-independent conditions

**Figure 5:**
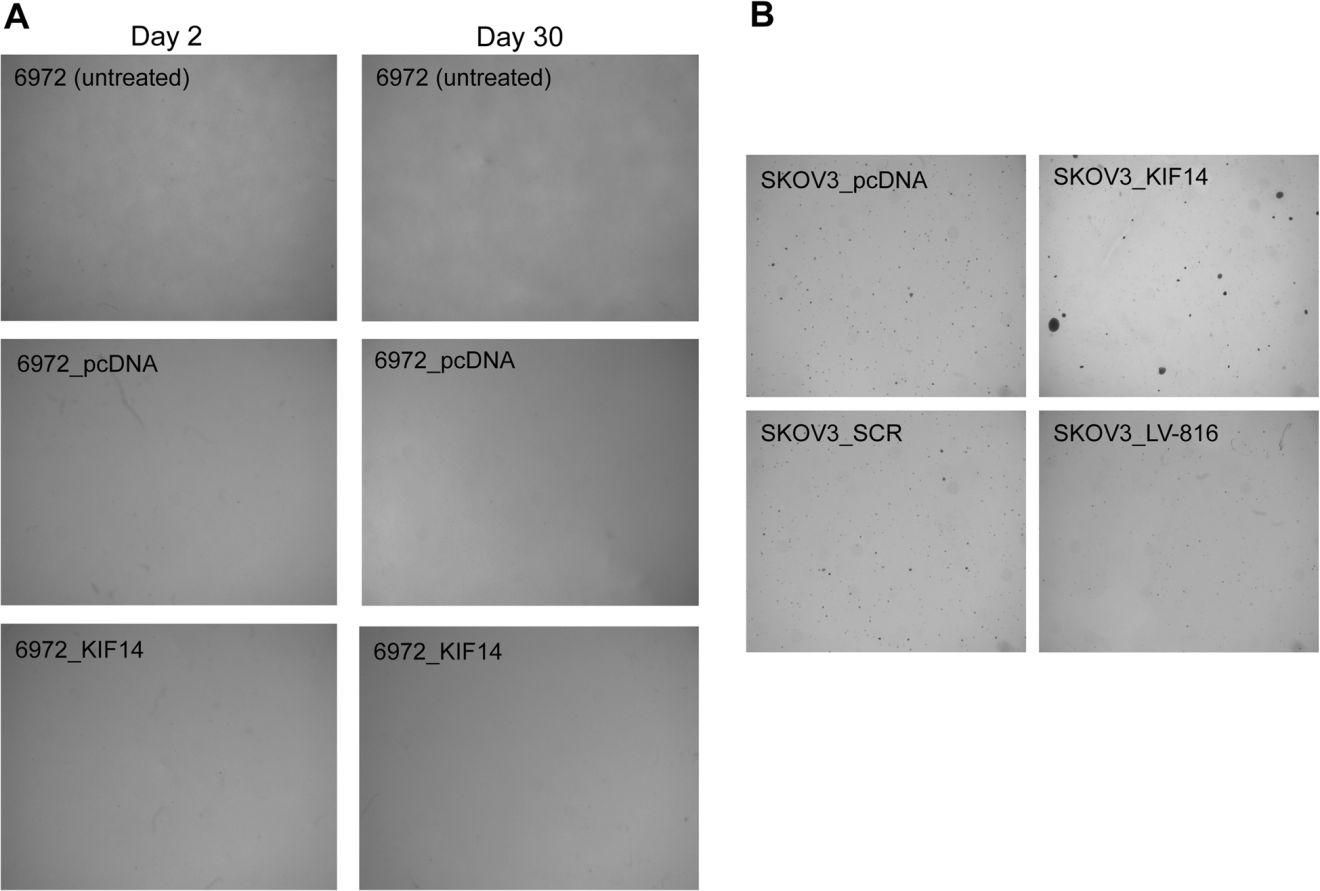
**Colony assays. A** Representative primary OvCa short-term cultured sample (6972) grown in soft agar either untreated (top), expressing control vector (middle) or overexpressing KIF14 (bottom panels). Images taken 2 and 30 days post seeding. **B** SKOV3 cells expressing either control vectors (pcDNA or SCR), overexpressing *KIF14* (SKOV3_KIF14) or knocked-down in *KIF14* (SKOV3_LV-816). Images taken 14 days post seeding. Magnification, 20X.

We evaluated *in vitro* anchorage independence via growth in soft agar. Unfortunately, none of the 11 primary samples grew under these conditions (whether transfected with KIF14-EGFP, transduced with LV-816, or their control counterparts (empty vector or scrambled control)), in comparison to the immortalized OvCa cell line SKOV3 (Figure 5). The assay was carried out for 90 days (typical soft agar assay is 14–30 days), however no growth was seen in the 11 primary samples even after this extended growth period.

For evaluation of *in vivo* growth, we chose 4 primary OvCa samples that demonstrated the highest overexpression and increase in proliferation (and the greatest decrease in expression/proliferation) in response to *KIF14* overexpression (or knockdown; samples 6972, 6973, 63639; Figure 3). We injected 1 million cells mixed with matrigel subcutaneously into NOD-SCID gamma (NSG) mice, and monitored growth over a 6 month period. Unfortunately, mirroring the results seen in soft agar, none of the 4 samples tested demonstrated any growth, including the cells overexpressing *KIF14*.

## Discussion

The prognostic potential of *KIF14* expression in OvCa [5] suggests a dependence on *KIF14* for tumorigenic behaviour. If high *KIF14* expression is sufficient to alter primary OvCa cell behaviour *in vitro* and *in vivo*, an oncogenic stimulus would be identified in OvCa, providing proof-of-concept for studying its tumor-promoting mechanisms. Seven out of the 11 samples tested demonstrated increased/decreased proliferation in response to *KIF14* overexpression/knockdown, indicating a dependence upon *KIF14* expression to maintain proliferative capacity. Four out of the 11 samples did not show a phenotypic response despite the fact that all samples showed significant overexpression of *KIF14* post-transfection, even after 21 days in culture (Figure 4). These results raise the possibility that there may be individual variability in the phenotypic responses to *KIF14* overexpression. Although not tested here, the types of molecular lesions contained within the primary tumors may partly explain this differential response. We have however shown that most of the primary samples demonstrate dependence on *KIF14* expression for proliferative capacity, indicating that *KIF14* may have a role in promoting OvCa cell survival and tumor growth.

Out of the 29 high-grade serous primary samples received, only 11 samples were developed into short-term cultures. The remaining 18 samples were processed, but no cells were outgrown/retrieved using our standard culture protocols [31]. This highlights the inherent difficulty in the development and maintenance of primary cultures. Barriers to successful culture include the state of the tumor tissue prior to processing, (necrosis, hypoxia, excessive tissue manipulations). Our culture protocols incorporate both a basic culture medium devoid of growth factors that could artificially promote cell growth, and a trypsinization procedure to remove fibroblast contamination – however some tumor cells may require the need of these to adapt and survive in a 2-dimensional culture environment [31]. None of the 11 samples tested demonstrated any growth in soft agar (11 samples). These results indicate that although we have shown that primary OvCa cells may depend on *KIF14* for *in vitro* proliferation, growth in anchorage-independent conditions cannot be supported in these primary short-term derived cultures.

There are several explanations for why these cells did not grow in agarose culture. One explanation is that the culture conditions to which the cells were initially exposed (even though for a relatively short period) changed the ability of these cells to survive in anchorage-independent conditions. Although we did not test the *in* vivo growth of freshly isolated tumor cells, adaptation of these cells to 2-dimensional adherent culture conditions may have altered their inherent tumorigenic behaviours and reduced the tumor-initiating capacity of these cells. Furthermore, as mentioned earlier, we found that short-term cultures of primary high-grade serous OvCa cells does result in decreased *KIF14* expression in comparison to their primary tumor tissue counterparts (Figure 1), indicating that our culture conditions can promote cell survival without the need of high oncogene activation (in this case, high *KIF14* expression). This could explain why in general, primary OvCa cells exhibited lower *KIF14* mRNA expression in comparison to primary tumor expression after short-term growth in culture.

Another point to consider is that our culture system does select for epithelial cell populations, and does not support the growth of stromal, mesenchymal or fibroblastic cells known to exist within the primary tumor microenvironment [31]; thus this pure epithelial cell population may demonstrate different tumorigenic behaviours as compared to cultures within a mixed population of cells. In fact, tumor-stromal cell interactions have been well documented to be crucial in pancreatic cancer cell growth and metastatic behaviours [32], and are also becoming increasingly important in the metastatic behaviour of primary ovarian cancers [33].

The four most proliferative cases were chosen to test the model using xenografts. Unfortunately none of these xenografts were successful in producing tumors. The type of xenograft that was chosen (subcutaneous xenograft) could have affected the survival of these cultured cells. Although tumorigenic growth was not observed using this xenograft method, other methods could be employed (intrabursal, mammary fat pad injections) to evaluate the tumorigenic capacity of these derived cell cultures. Alternatively, unpublished data suggest that prolonged exposure to *in vitro* growth conditions prevents growth in xenografts. To date, no publication has described xenografts derived from primary cells grown in culture, which represents a major obstacle to this type of research.

Our results indicate that although increases in proliferative capacity of short-term cultured cells derived from OvCa patient tumors were seen with *KIF14* overexpression, other changes in carcinogenic signalling pathways may also be required for survival and growth of these cells in anchorage-independent conditions. In other words, in primary tumor cells which possess limited genomic changes, *KIF14* overexpression may not be sufficient to induce tumor growth *in vivo*, as compared to established cell lines, known to have high genomic instability and possess the capacity to form anchorage-independent colonies and form subcutaneous tumors. It would be interesting to evaluate whether combining *KIF14* overexpression with other known genetic and molecular perturbations present in OvCa (p53, PTEN, PI3K, MAPK) [34,35] would affect the anchorage-independent properties of these cells.

In summary, we were able to evaluate the effect of *KIF14* manipulation on the *in vitro* growth of short-term primary OvCa cultures. We determined that most of our short-term derived primary OvCa cultures were dependent on *KIF14* expression for growth *in vitro*, but that for a number of technical reasons, these cells could not demonstrate growth *in vivo.* We have thus demonstrated the development of a useful and effective *in vitro* system of gene manipulation to assess growth properties of primary OvCa cells, successfully evaluating the phenotypic effect of a potential oncogene on proliferative capacity. This method could also be employed to evaluate responsiveness to therapeutic interventions.

### Highlights

• We evaluated the potential of selective *KIF14* inhibition in primary high-grade serous patient-derived OvCa cells

• Short-term cultures of primary high-grade serous demonstrated dependence upon *KIF14* expression to maintain proliferative capacity

• An effective *in vitro* method to evaluate target gene manipulation on the proliferative capacity of primary OvCa cultures was developed.

## Additional files

Additional file 1: Figure S1. Expression of KIF14 in primary OvCa cultures in response to anti-KIF14 shRNA lentivirus transduction. **A** Representative sample (6973) was imaged for KIF14 expression using immunofluorescence microscopy following transduction with either a scrambled shRNA control (SCR; top panel) or an anti-*KIF14* shRNA (LV-816; bottom panel). Images taken at 14 days post-transduction. Cells were stained with anti-KIF14 antibody (green, left panels) or DAPI to reveal nuclei (blue, right panels). Magnification, 400X. **B** Representative immunoblot of 6973 cells transduced with anti-*KIF14* shRNA (LV-816-1 and −2; 1 and 2 represent 2 different transduction experiments), with scrambled shRNA (SCR) or untransduced (U), assayed 14 days post-transfection. β tubulin, loading control. Additional file 2: Figure S2. Expression of EGFP tag in primary OvCa cultures in response to KIF14 transfection. **A** Representative sample (6972) was imaged for KIF14 expression using fluorescence microscopy following transient transfection with either an empty vector control (pcDNA; top panel) or a KIF14-EGFP-tagged construct (KIF14-EGFP; bottom panel). Images taken at 14 days post-transfection. Cells were stained with anti-KIF14 antibody (top left panel), or visualized by EGFP fluorescence (bottom left panel), and stained with DAPI to reveal nuclei (blue, top/bottom left panels). Magnification, 400X. **B** Representative immunoblot of 6972 cells transfected with KIF14-EGFP (KIF14-1 and −2; 1 and 2 represent 2 different transfection experiments), with empty vector (pcDNA) or untransfected (U), assayed 14 days post-transfection. β tubulin, loading control.
