# Supplementary figures and images for: The role of KIF14 in patient-derived primary cultures of high-grade serous ovarian cancer cells
Source: J Ovarian Res. 2014 Dec 21;7:123. doi: 10.1186/s13048-014-0123-1 (PMC4302703; doi:10.1186/s13048-014-0123-1)

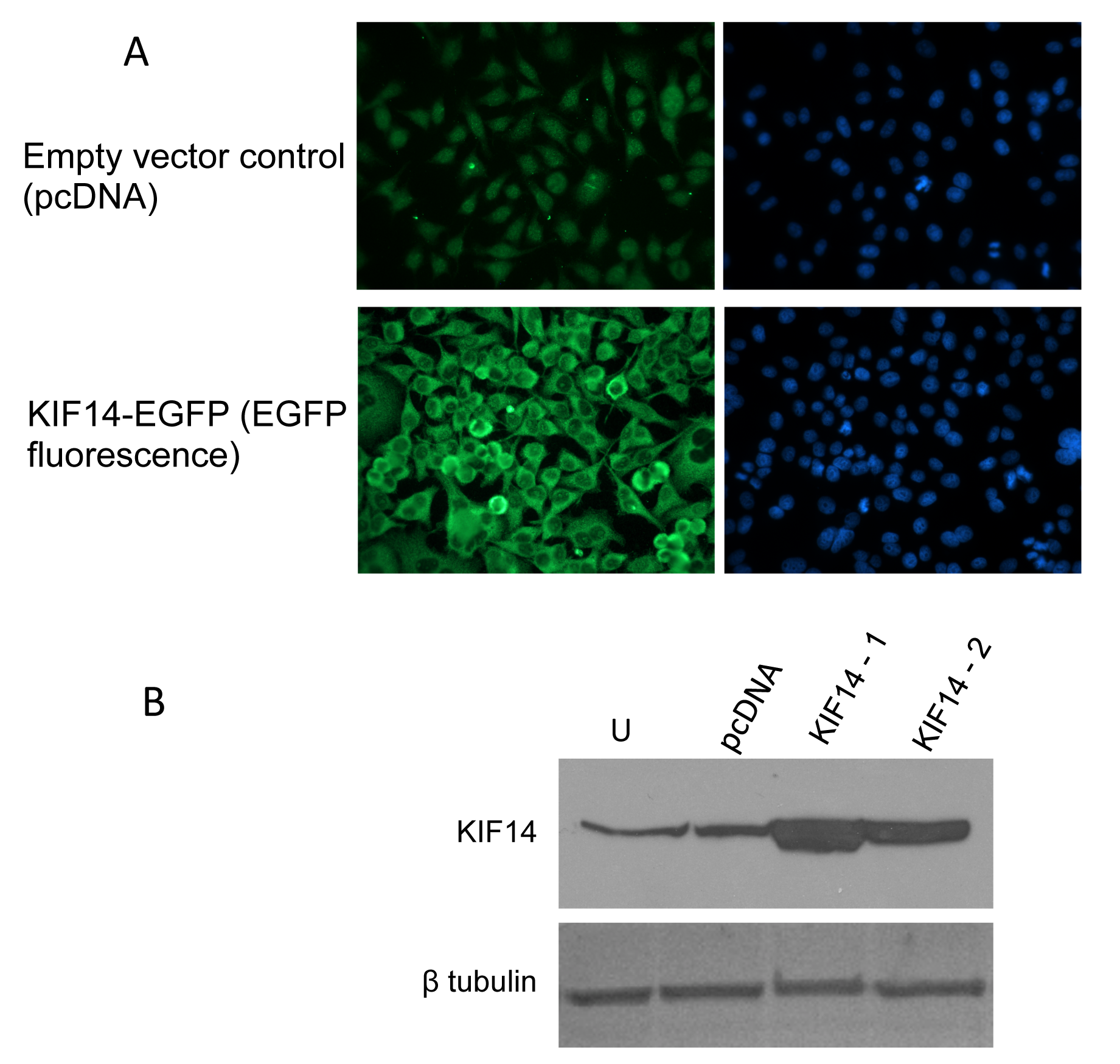

Supplement: Additional file 1: Figure S1. — Expression of KIF14 in primary OvCa cultures in response to anti-KIF14 shRNA lentivirus transduction. A Representative sample (6973) was imaged for KIF14 expression using immunofluorescence microscopy following transduction with either a scrambled shRNA control (SCR; top panel) or an anti-KIF14 shRNA (LV-816; bottom panel). Images taken at 14 days post-transduction. Cells were stained with anti-KIF14 antibody (green, left panels) or DAPI to reveal nuclei (blue, right panels). Magnification, 400X. B Representative immunoblot of 6973 cells transduced with anti-KIF14 shRNA (LV-816-1 and −2; 1 and 2 represent 2 different transduction experiments), with scrambled shRNA (SCR) or untransduced (U), assayed 14 days post-transfection. β tubulin, loading control. [file 13048_2014_123_MOESM1_ESM.tiff]

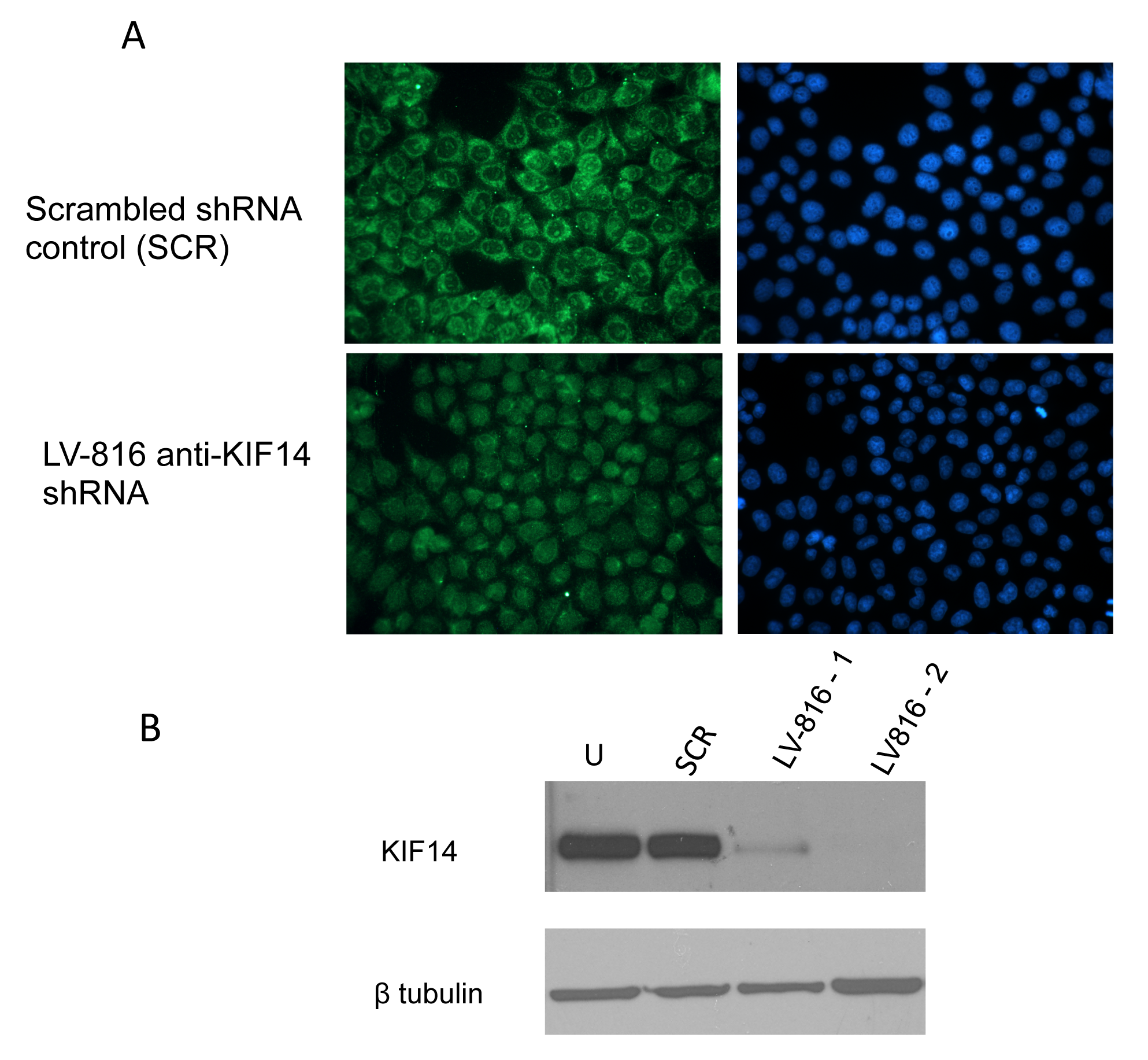

Supplement: Additional file 2: Figure S2. — Expression of EGFP tag in primary OvCa cultures in response to KIF14 transfection. A Representative sample (6972) was imaged for KIF14 expression using fluorescence microscopy following transient transfection with either an empty vector control (pcDNA; top panel) or a KIF14-EGFP-tagged construct (KIF14-EGFP; bottom panel). Images taken at 14 days post-transfection. Cells were stained with anti-KIF14 antibody (top left panel), or visualized by EGFP fluorescence (bottom left panel), and stained with DAPI to reveal nuclei (blue, top/bottom left panels). Magnification, 400X. B Representative immunoblot of 6972 cells transfected with KIF14-EGFP (KIF14-1 and −2; 1 and 2 represent 2 different transfection experiments), with empty vector (pcDNA) or untransfected (U), assayed 14 days post-transfection. β tubulin, loading control. [file 13048_2014_123_MOESM2_ESM.tiff]
